# Supplementary material for: Venomix: a simple bioinformatic pipeline for identifying and characterizing toxin gene candidates from transcriptomic data
Source: PeerJ. 2018 Jul 31;6:e5361. doi: 10.7717/peerj.5361 (PMC6074769; doi:10.7717/peerj.5361)
Supplement: Supplemental Information 3 [file peerj-06-5361-s003.gz › FinalOutput_E-20/Snaclec_VP12_subunit_A_1/finaltree.pdf]

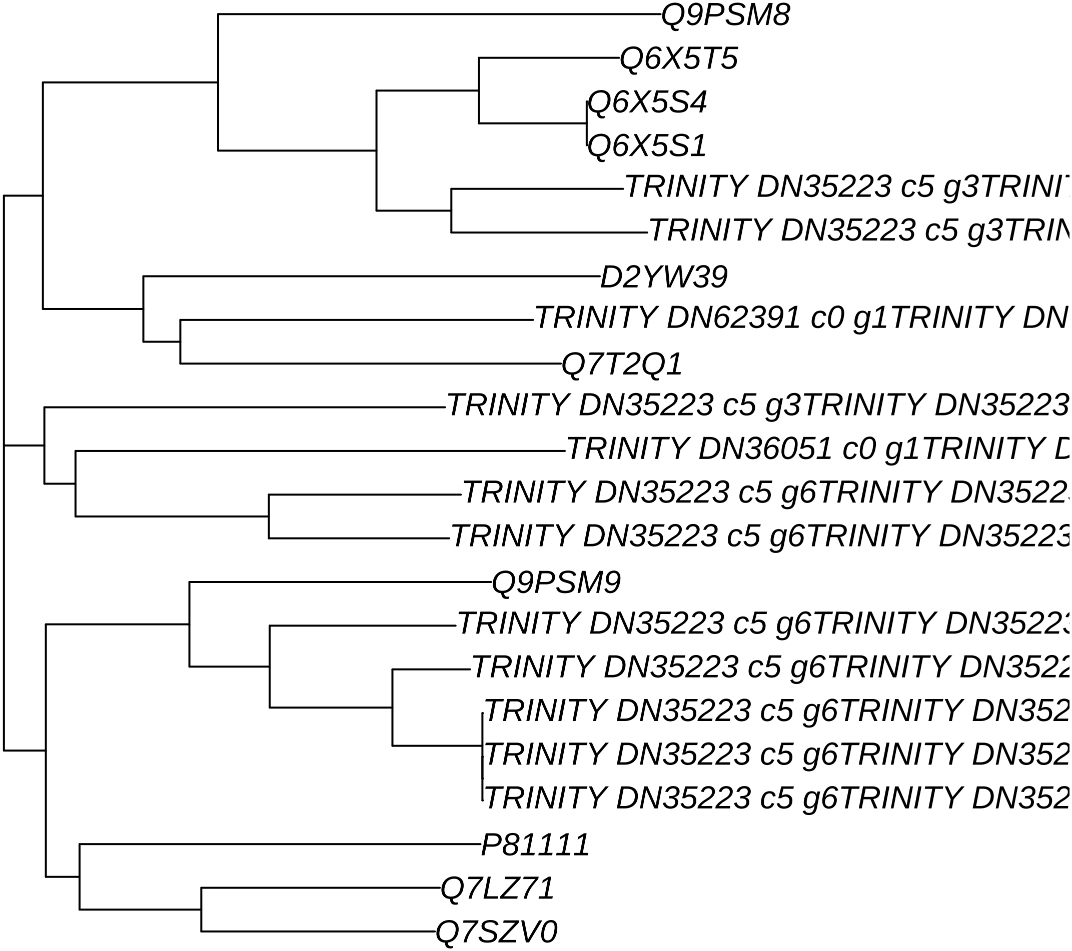
Q9PSM8
Q6X5T5
Q6X5S4
Q6X5S1
TRINITY DN35223 c5 g3TRINIT
TRINITY DN35223 c5 g3TRIN
D2YW39
TRINITY DN62391 c0 g1TRINITY DN
Q7T2Q1
TRINITY DN35223 c5 g3TRINITY DN35223
TRINITY DN36051 c0 g1TRINITY D
TRINITY DN35223 c5 g6TRINITY DN3522
TRINITY DN35223 c5 g6TRINITY DN35223
Q9PSM9
TRINITY DN35223 c5 g6TRINITY DN3522
TRINITY DN35223 c5 g6TRINITY DN3522
TRINITY DN35223 c5 g6TRINITY DN352
TRINITY DN35223 c5 g6TRINITY DN352
TRINITY DN35223 c5 g6TRINITY DN352
P81111
Q7LZ71
Q7SZV0
